# Health literacy experiences of multi‐ethnic patients and their health‐care providers in the management of type 2 diabetes in Malaysia: A qualitative study

**DOI:** 10.1111/hex.13095

**Published:** 2020-07-19

**Authors:** Adina Abdullah, Su May Liew, Chirk Jenn Ng, Subashini Ambigapathy, P. Vengadasalam V. Paranthaman

**Affiliations:** ^1^ Department of Primary Care Medicine University Malaya Primary Care Research Group (UMPCRG) Faculty of Medicine University of Malaya Kuala Lumpur Malaysia; ^2^ Health Clinic Buntong Ministry of Health Malaysia Ipoh Malaysia; ^3^ Health Clinic Greentown Ministry of Health Malaysia Ipoh Malaysia

**Keywords:** health literacy, Malaysia, qualitative research, type 2 diabetes mellitus

## Abstract

**Background:**

Patients with type 2 diabetes mellitus (T2DM) require adequate health literacy to understand the disease and learn self‐management skills to optimize their health. However, the prevalence of limited health literacy is high in patients with T2DM, especially in Asian countries.

**Objective:**

This study aimed to explore experiences related to health literacy in Asian patients with T2DM.

**Design:**

This is a qualitative study using in‐depth interviews and focus group discussions. A framework analysis was used to analyse the data.

**Setting and participants:**

articipants (n = 24) were multi‐ethnic patients with T2DM (n = 18) and their primary health‐care providers (n = 6). This study was conducted in four primary health‐care clinics in Malaysia.

**Results:**

Nine subthemes were identified within the four dimensions of health literacy: accessing, understanding, appraising and applying information.

**Discussion:**

Motivated patients actively sought information, while others passively received information shared by family members, friends or even strangers. Language and communication skills played important roles in helping patients understand this information. Information appraisal was lacking, with patients just proceeding to apply the information obtained. Patients' use of information was influenced by their self‐efficacy, and internal and external barriers.

**Conclusion:**

In conclusion, the experiences of multi‐ethnic patients with T2DM regarding health literacy were varied and heavily influenced by their cultures.

## 1 INTRODUCTION

Health literacy is an important ability for patients when negotiating complex and long‐term health conditions such as type 2 diabetes mellitus (T2DM).^1^ It is defined as the degree to which individuals can access, understand, appraise and apply health information to make informed health decisions to maintain quality of life across their life course.^2^ It is closely related to literacy and entails patients' knowledge, motivation and competences.^2^ Health literacy is of particular importance to patients with T2DM to enable the use of information and services to make appropriate lifestyle and treatment decisions.^3^

Studies have shown that limited health literacy in patients with T2DM was associated with adverse health outcomes. These patients had difficulty in reading printed instructions and understanding health recommendations or warnings.^4^ They were also reported to have less disease knowledge,^5^, ^6^ poorer medication adherence^7^ and expended more money on health care.^8^ These patients also experienced poorer patient–doctor communications and participated less in shared decision making.^9^ However, evidence linking limited health literacy and glycaemic control is more mixed. Some studies have demonstrated links between higher levels of health literacy and lower HbA1c, while others have failed to show an association.^5^, ^10^, ^11^, ^12^

Despite the mixed findings, diabetes self‐management interventions that considered health literary aspects showed positive effects on glycaemic control. In 2016, a systematic review and meta‐analysis of health literacy‐sensitive diabetes self‐management interventions found a significant reduction in HbA1c for health literacy‐sensitive interventions when compared to usual care; the effect was more pronounced in patients with limited health literacy.^13^ Studies that employed interventions focusing on self‐management in patients with limited health literacy also found a significant reduction in emergency department visits and hospitalizations.^14^ Many of these interventions were educational, employing the use of written communication, oral communication, patient empowerment and tailored communication to patients' language and cultural practices.^13^

Limited health literacy in patients with T2DM is prevalent, and the rate is even higher in Asian countries with multi‐ethnic populations. A recent systematic review found the pooled prevalence of limited health literacy in the United States of America to be 30% and the prevalence in European countries to range from 7.3% in Switzerland to 9.7% in the Netherlands.^15^ In Asian countries with multi‐ethnic populations, the prevalence ranged from 71.7% in South Korea to 82% in Taiwan.^4^, ^16^ In Malaysia, recent studies have shown the prevalence of limited health literacy in patients with T2DM to be as high as 85.8%.^17^

Recent qualitative studies exploring health literacy in patients from Asian cultures found that culture shaped patients' understanding and experiences of health literacy. In a study exploring health literacy experiences of Chinese patients with diabetes living in America, Leung et al^18^ found that cultural issues influenced patients' access, understanding and application of information. Similar findings were noted in Thai patients with diabetes,^19^ and in Samoa, the health literacy of patients with non‐communicable diseases is heavily influenced by culture.^20^

Malaysia is an ideal location to study the health literacy experiences of multi‐ethnic Asian patients. Malaysia is a multi‐ethnic, multi‐cultural and multi‐lingual country where the major Asian ethnic groups Malay, Chinese and Indian still maintain somewhat separate cultural identities. Malaysian society had been described as ‘Asia in miniature’.^21^ Furthermore, T2DM is a major health problem in Malaysia. The prevalence of diabetes in Malaysia was 17.5% in 2015.^22^ Only 70% of these patients achieved target glycaemic control, thus leading to a high prevalence of microvascular and macrovascular complications.^23^, ^24^ Therefore, this study aimed to explore health literacy experiences in the management of T2DM in multi‐ethnic patients and their health‐care providers (HCPs) in Malaysia.

## 2 METHODS

### 2.1 Design

This study used a qualitative approach where in‐depth interviews and focus group discussions were conducted to explore the experiences of multi‐ethnic patients with T2DM and the primary HCPs who supported them.

### 2.2 Setting

This study was conducted in four primary health‐care clinics in the state of Perak, Malaysia, from January to April 2019. Participants were patients with T2DM previously involved in a study measuring the prevalence of limited health literacy at the same setting. They were purposively selected to represent the experiences of patients with adequate and limited health literacy. Health‐care providers (HCPs) who participated in the study worked at the study clinics and were involved in delivering care to the patients with T2DM involved in this study. Most patients approached agreed to be interviewed, three patients declined, two had relocated and one was too ill. All HCPs approached agreed to be interviewed.

### 2.3 Topic guide

The conceptual model of health literacy by Sørensen^2^ guided the development of the topic guide.^2^ Questions were kept as open‐ended as possible with the aim of exploring participants' views and experiences within the four dimensions of health literacy. Separate topic guides were created for patients' interviews and for HCPs' interviews. The topic guide for patients was developed in English and then translated to Bahasa Malaysia and Mandarin, whereas that for HCPs was in English. The topic guide was revised after each interview to include new issues raised by the participants. There were no new revisions of the topic guide after three patient interviews and two HCP interviews. The topic guides for patient and HCP interviews were included as Appendix S1 and S2.

### 2.4 Sampling and data collection

Patients were recruited through phone calls, and the HCPs were recruited by SMS messages (WhatsApp) and email. All participants were provided with the participant information sheet and given time to read through it before the interview. Those who agreed to participate were then asked to sign a consent form. All interviews were conducted in a private room at the study sites. Interviews in English and Bahasa Malaysia were conducted by the first author (AA). A trained research assistant (CHY) conducted the Mandarin interviews. CHY was present at three of the interviews conducted by AA and took notes for both focus group discussions. The interviews were audio‐recorded. There were 18 patients' in‐depth interviews, one HCP in‐depth interview and two focus group discussions involving five HCPs.

### 2.5 Data analysis

The interviews were transcribed verbatim, and the data were managed using ATLAS.ti 9 software. Transcripts in other languages were translated and analysed in English. Interviewers' reflective notes were written after the in‐depth interviews, and field notes were taken during the focus group discussions. Each in‐depth interview lasted for 30‐45 minutes, and the focus group discussions lasted for 45‐60 minutes each. Data reached saturation after the 16th patient interview. The HCP interviews did not reach data saturation, but these interviews were performed to triangulate patients' data. Transcriptions were done by professional transcribers and checked by the first author by listening back to the audio recordings of the in‐depth interviews (IDIs) and focus group discussions (FGDs).

The data were analysed using the five stages of the thematic analysis framework: (a) familiarization; (b) identifying a thematic framework; (c) indexing; (d) charting; and (e) mapping and interpretation.^25^ Familiarization involved gaining an overview of the literature, research objectives and data. Then, a framework was identified. In this study, in order to explore all the capacities associated with health literacy, the thematic framework was informed by Sørensen's^2^ conceptual model of health literacy. The domains of health literacy made up the categories for data analysis.

All interview transcripts were then systematically coded using the thematic framework created. All the coding was completed by AA, and LSM and NCJ double‐coded two transcripts. The researchers involved in the analysis were primary care physicians (AA, LSM and NCJ) who were conscious of their personal views and biases concerning health literacy in patients with T2DM. Quotations that best reflected the themes that emerged from the transcripts are presented in results.

The rigour of this research was ensured by using the criteria of credibility and confirmability. In order to achieve credibility, the topic guides were developed using an established conceptual framework and data triangulation was performed by using two data collection techniques, that is IDI and FGD, as well as collecting data from patients and their HCPs to capture patients' experiences related to health literacy from two different perspectives. Transcripts of the interviews were also shared and checked with the participants in a process called member checking. Researcher bias was addressed by the main researcher completing a reflective diary at the end of every interview, which captured the researcher's perceptions and opinions, which were then bracketed during data analyses. Dependability was achieved by having two other researchers read through the transcripts and agree on the codes and themes generated.

## 3 RESULTS

The interviews yielded rich data pertaining to the way patients access, understand, appraise and apply health information in the management of their T2DM. The participants' characteristics and study sites are summarized in Table 1. Categories, themes and subthemes are set out in Table 2. Quotations are labelled with the prefix P for participant followed by the participant's number, for example P1 denoting participant 1.

**TABLE 1:** Characteristics of participants (n = 24)

Patients (n = 18)	Study site	Age	Ethnicity	Gender	Health literacy (HL) level
Participant 1	Clinic 1	56	Indian	Man	Limited HL
Participant 2	Clinic 1	56	Indian	Woman	Limited HL
Participant 3	Clinic 4	58	Indian	Man	Adequate HL
Participant 4	Clinic 4	56	Malay	Man	Adequate HL
Participant 5	Clinic 4	56	Chinese	Woman	Limited HL
Participant 6	Clinic 2	59	Chinese	Woman	Adequate HL
Participant 7	Clinic 2	58	Malay	Woman	Adequate HL
Participant 8	Clinic 2	59	Malay	Woman	Adequate HL
Participant 9	Clinic 2	59	Malay	Man	Limited HL
Participant 10	Clinic 1	58	Punjabi	Woman	Limited HL
Participant 11	Clinic 1	56	Indian	Woman	Adequate HL
Participant 12	Clinic 3	58	Malay	Man	Adequate HL
Participant 13	Clinic 3	56	Malay	Man	Adequate HL
Participant 14	Clinic 3	59	Malay	Woman	Adequate HL
Participant 15	Clinic 3	58	Malay	Man	Limited HL
Participant 16	Clinic 3	59	Malay	Woman	Limited HL
Participant 17	Clinic 1	57	Chinese	Woman	Limited HL
Participant 18	Clinic 4	57	Chinese	Man	Limited HL
Health‐care providers (n = 6)	Study site	Age	Ethnicity	Gender	Positions
Participant 19	Clinic 1	36	Chinese	Man	Family physician
Participant 20	Clinic 1	52	Indian	Man	Medical officer
Participant 21	Clinic 1	32	Indian	Woman	Medical officer
Participant 22	Clinic 4	37	Indian	Woman	Diabetes educator
Participant 23	Clinic 2	43	Malay	Woman	Diabetes educator
Participant 24	Clinic 3	37	Malay	Woman	Diabetes educator

**TABLE 2:** Categories, themes and subthemes

Categories	Themes	Subthemes
Access/finding information	Formal and informal sources	HCP's responsibility to give information
		HCP as important source of information
		Family, friends and community
	Push and pull information	Testimony from friends and family—especially from those with diabetes
		Lack of skills to find information on their own
	Motivation	Empowered patients seek information
		Unmotivated to look for information
Understand information	Language	Language helps understanding
		Information delivered in DSME easy to understand
	Communication and clarification skills	Need more clarifications
Appraise information	Trust and belief	Based on trust
		Based on logic and personal belief
		Based on the consistency of information across websites
Apply information	Self‐efficacy	Self‐efficacy
	Barriers	Personal and system barriers

### 3.1 The health literacy experiences of multi‐ethnic patients with T2DM

**FIGURE 1:**
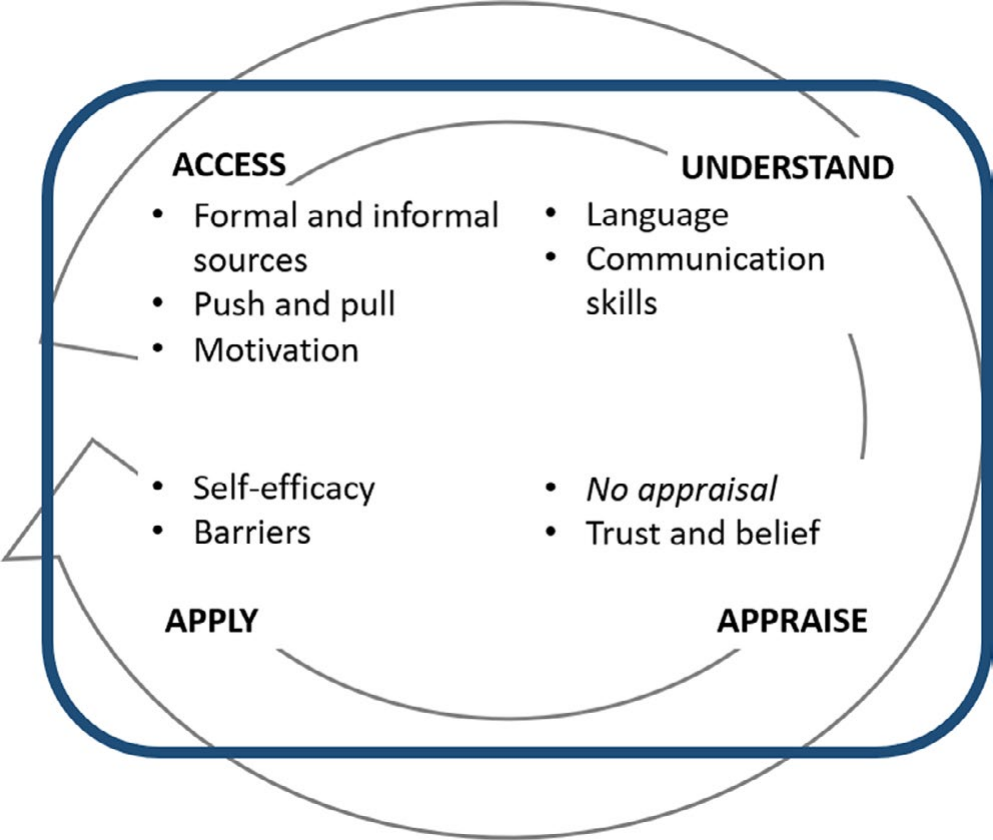
Factors influencing health literacy in multi‐ethnic patients with T2DM

Patients with T2DM and their HCPs shared their health literacy experiences, and these were then coded into the four health literacy domains of accessing/finding, understanding, appraising and applying information. For each domain, subthemes were identified to represent factors that influence participants' experiences (Figure 1). Direct quotations from participants' experiences were used to illustrate the findings.

### 3.2 Accessing/finding information

We found three subthemes relating to participants' experiences in accessing/finding information: formal and informal sources of information, push and pull information, and motivation.

#### 3.2.1 Formal and informal sources

Patients sourced their information from informal and formal sources. The main formal source was their HCPs, particularly doctors. Participants felt that it was the doctors' responsibility to provide them with information and that ‘if the doctor did not tell, I won't know’:

It is their (doctor's) responsibility. It is their responsibility to give advice, advice, advise people. (P4)

When I came here…the doctor told me,…told me about diabetes, aaaa…about the effect of the medicine, that's how I was enlightened, by coming here. (P15)

(Ohhh can, oo okay, if the doctor did not tell you, what will happen?)

If the doctor does not tell me, I will just continue to take the medicine, I won't know. (P16)

This belief stemmed from their lack of skills in looking for information themselves or because they felt doctors had more expertise in this area:

Sources from the doctors. Yes, because doctors have a better understanding (in this matter). (P12)

Where to find (this information)? How would I know? If the doctor tells me, (laughing) then I would know. (P16)

The main issue participants noted was that HCPs were only interested in sharing information if the disease was uncontrolled. Participant 12 noted that his doctors did not give him much information because his diabetes was under control:

Err…. Maybe because the sugar level didn't go up, so whatever still maintain, so like the doctor doesn't, you know, doesn't tell you anything, and unless if maybe if the level goes up, then maybe… maybe they will discuss about it. (P6)

I guess my condition wasn't too serious yet. If it were serious, he would say something. (P12)

Participants' informal sources of information were from their family members and friends, especially from those who were also diagnosed with T2DM:

She said, she (my sister) said we have diabetes so have to be careful, first of all do not get any wound. She said we need to wear those shoes, she said if possible, the shoes that cover your toes, not exposed… she is worried that we may get hurt, this is the danger of diabetes. (P7)

Hmmm…yes I know, because my husband also err… has diabetes. (P6)

HCPs understood their responsibility to share information with patients but felt they should also empower patients to look for information themselves. HCPs admitted that they only gave advice to patients with poor control, as a lack of consultation time limited the number of patients and the depth of discussion that they could engage in:

So, we play a very important role to educate. And I think we should also actively provide sources of information, ya. (P19)

We cannot be telling everything because of time again. If anything is abnormal then we will bring it up. Otherwise, if everything is okay, we don't discuss it anymore. (P20)

#### 3.2.2 Push and pull information

As opposed to accessing information by themselves (pull), participants told us that information was also ‘pushed’ to them. When patients were actively searching for information to assist in their effort to manage their diabetes, information was being pulled by patients from various sources as discussed above. However, there were instances where information was shared with patients whether they solicited it or not:

Anybody where… anyone wherever we go, sometimes we go (to) temple will talk… (they will say) this one got diabetes, they said okay, you all take this take that. (P10)

I heard it here (at the clinic), there were these ladies talking, I just listened. I don't really like to engage in conversations, just listen. (P8)

HCPs observed that only motivated patients would look for or pull information, whereas others became more confused with information shared by others or developed stigma from information shared by others:

If they are really motivated, can . Maybe the younger I don't think not the, ours is senior citizens onwards, so they just come, they just go. (P20)

From outside (of clinic), and their belief is set on saying this diabetes drug causes kidney failure. So, it is very hard for us to fix, aa to fix their myths. (P24)

#### 3.2.3 Motivation

Patients who went out to search for information were those who were motivated to do so and could appreciate the value of such pursuit. Some had seen the suffering of other patients with T2DM and believed that with appropriate information they could better control their illness:

For us diabetics must be the individual, the one with the illness, the sick, the sick themselves. (P4)

Because I see this person suffering from diabetes. suffer … my friend has diabetes, and heart disease. She didn't go to the doctor for check‐ups and most of the people I asked didn't see the doctor for regular medication. So, I would rather learn from the beginning. (P15)

In contrast, there were participants who did not see any value in looking for information. They believed in fate and did not have any adverse symptoms from the disease and thus were not motivated to look for information. Some even felt scared to look for information which might tell them of the potential negative outcomes of the illness:

(Laughing) Don't know, it is Allah's job to hold our lives. If He wanted to take it, He will take it….(P9)

No no, I have diabetes (if) my diabetes went up I will know it. My whole body shakes. (P1)

Ah, so we did, we felt scared when we heard it…read it…. Of course. You heard someone has to cut off their feet, right? Ah, if there is a wound, right? It's hard to heal, right? I got scared. (P8)

### 3.3 Understand information

Subsequent to finding information, participants shared their experiences in understanding the information they found. We found two subthemes in this domain: the influence of language, and communication and clarification skills.

#### 3.3.1 Language

Patients said that English is the language of science, but those who were not fluent in English sought information in the language in which they were fluent. They even looked for doctors who were able to converse in their mother tongue:

English………the language of science. (P4)

(the information on) the Internet that I search, it is in Chinese, in Chinese … (if not) difficult …. I don't understand English. Hmm, when I went to have my health check at (the clinic), eh I will also look for a Chinese doctor …. (P5)

Doctors, they will actually look at the patient, if the patient could converse in English, they will talk in English , if they cannot, they will speak in Malay ….(P4)

Some patients used Internet tools or the dictionary to support their understandings. Some used the translate function on their Internet browser to translate the information to the language they understand:

Can, can, I can also read English. But both (English and Malay) also can. But with Google now easy, it can translate for you. (P13)

Hm for me…I can't read English very well. There are words, err…words that are very difficult for me to understand, I look up in the dictionary. (P2)

Patients found information delivered at the diabetes self‐management education sessions easy to understand:

But nothing is too difficult. They just tell you simple instructions… don't eat rice at night, exercise. It's quite easy to understand. (P4)

It's not that hard to follow actually. It really depends on you whether you want to follow or not. Eat less rice, eat more vegetables. That's why I said, it's not that hard. (P14)

HCPs agreed that language helped understanding—they gave examples of where they found that the ability to speak in a language other than English or Malay helped them communicate better with their patients:

Ok so my, my, my medical officers you know, they refer Chinese patients to me. Some of the Chinese patients regardless whether they are young or old, some of them just, just cannot communicate or understand Bahasa Malaysia. (P19)

To make them (patients) understand, doctor. To make them understand is a challenge, especially regarding language. That is my challenge, I have to learn Chinese. (P22)

#### 3.3.2 Communication and clarification skills

When patients gathered information independently, they found that they had unanswered questions that required clarification. Some patients tried to engage the HCPs to assist them:

Ok. I think, can the medication be stop actually or not? Sometimes I got, erm… actually I do try to skip also sometimes, I don't really like to follow everyday one metformin. I do like (take it) on and off. (Did you mention this to your doctor?) Err… I think I did, I did, but they said continue and said maybe your level is like that because you are on the medication. So that's what they said. (P6)

Normally after I eat, around 6pm I will inject the insulin, after injection it was fine, but I will wake up in the middle of night sweating, isn't it strange? How can my blood glucose level go up and down? It should be high. (P18)

No, err… like the what… actually ya, you can just tell me a little bit like actually what we must eat, how, how, but I eat Capati (flat bread) in the morning, yes I take one Capati (flat bread), afternoon I take one and a half, at night I take one. (P10)

HCPs, especially diabetes educators, noted that patients often asked them about new drugs they had come across in mass media (in the news). However, doctors believed that patients needed to be empowered before they could engage them in discussions to tackle any misconceptions:

So, for example, they have never taken (a new diabetes medication). But they will ask er is (that medication) good, why did the doctor not give it? Why did the doctor give insulin? (P23)

Aa, probably we have not empowered them enough to, to learn to er clarify the information, you know. Er some people say that, you know some of my patients that I've encountered saying that you know, using insulin can cause renal impairment, alright. So I have, after interview with them, you know I realise, realise that they actually have a lot of misconceptions. (P19)

### 3.4 Appraising information

Participants moved from accessing/finding and understanding information straight to applying information. They did not engage in evaluation of the information prior to applying it:

I believe more in the information regarding medications and diet only. The other information, I take them less seriously. I am also not sure which is right or wrong. Yes, I just accept. If I can follow, I will follow. If I can't then I will just forget it. (P12)

HCPs also made the same observation. They felt patients did not reflect on what was given to them and were more likely to just follow the instructions given by HCPs:

For the older generation er they tend to just get from the information and they just take in the information, they did not er you know, clarify or, or criticise whether the information that they received is accurate or not. They believe it as a gospel truth. (P19)

Ya, actually it's a time consuming , actually they don't understand why we are doing so just go only, ECG , check , eye check , they don't know what's happening, they may go to dietician la, maybe the dietician a little bit quite going , actually they do not, don't know anything also, they come for the follow‐up, they just follow what they are supposed to do, take the medicine properly, don't need to know anything one. So, better , Ok. (P20)

#### 3.4.1 Trust and belief

Much of the information was gathered orally by approaching their HCPs, families and friends (principally those with diabetes). These were people who they trusted to give them the right information. Therefore, trust played an important role in their decision to believe the information or not:

Er … if he is, if he is my friend. We always meet up. I have four friends, two have diabetes both of them. I totally believe them; I can see what they say…I just follow. (P3)

I trust the doctor more than friends. Because the doctor studies some people just ask their friends. This root, this shoot, just take it? Doctors learn the doses of things. (P9)

Patients also trusted information found online, because they believed that the government regulated this information and that most of it was written by HCPs. They also believed that if the information was consistent across many websites or repeated by different doctors, then it must be true:

(Information) that is on the Internet can be trusted. Maybe the information is written by doctors, the source of the information is from doctors also…now information is at your fingertips. (P15)

If it's on the Internet, then it should be harmless right, they are regulated by the government. (P18)

The info, most of the websites, the info is about the same. So, I… I trust that it is true. (P6)

But for me…I don't just refer to one source, right? Maybe two or three. And if they (different sources) state the same information, then it must be right. (P13)

One patient observed that neither the HCPs nor complementary and alternative (CAM) practitioners shared or discussed evidence with him. He then said it was very difficult to evaluate which information was true that he received from the HCP or the CAM practitioner:

Hmmm…ehem … so I…I don't see who…hmm what a friend says, there is no evidence. The doctor also gave no evidence so I (am stuck) in the middle, the doctor said if I don't (take the medicine) I will have kidney disease. Where is the evidence, there is none. Other people say if you take too much medications you will get kidney disease, also no evidence…hmmm so I'm confused. (P15)

### 3.5 Applying information

In the final stage of their health literacy journey, patients shared their experiences of applying health information. Self‐efficacy seemed to be the main influencing factor here, but patients did discuss the personal and environmental barriers faced when applying information.

#### 3.5.1 Self‐efficacy

Patients in our study declared that the decisions to act on the information were their own. They had ownership of their lives and health, as one participant stated, ‘diabetes is my life’ (P3):

Not really, it starts with your own self, doesn't it? Like the proverb ‘when there is a will, there is a way’. (P7)

It's good that we want to do it ourselves. If we love our bodies, our lives, we do it. If you don't love it, love it. (P4)

Err…. Yes, ya. I think that is the only way where you think you know you want to be better definitely you have to get info from somewhere isn't? And I do follow what they say, ok. (P6)

HCPs found self‐efficacy to be the main factor for patients to act on information given to them. They also noted some internal and external barriers faced by patients:

Er Ok most patients actually I think they can understand our instructions and all but some they find it difficult to follow, I'm not sure why, maybe because of their work, er some they are working shift hours then it is difficult to take their medicine or sometimes they often forget,…diet control is definitely (chuckles) quite bad. (P21)

#### 3.5.2 Barriers

Patients also shared the barriers they faced when trying to apply health information. These barriers were related to personal barriers and environmental barriers. Patients cited lack of self‐control and self‐discipline as internal barriers, and time and family responsibilities as external barriers:

Aa that (laughs) people like to give (sweet food) to me. My heart says no, but I still want them (laughs). (P1)

Yes. I cannot resist the temptation. I felt like I can still eat unhealthy food at small amount, then slowly the amount increased. (P18)

12 hours. I work as a receptionist. In my work I sit a lot in air‐conditioned room, right? So, when do I have time to exercise? Start work at 6.30 am and finish at 6.30 pm. When I go home at 6.30 pm, I am very tired. I only have one off day, that is the only day I can rest and do a lot of errands…to exercise is…less… (P7)

In contrast, some patients said their family members and friends assisted in the implementation of the information gathered. Family members often reminded patients to implement the information given or gathered. They also helped assist patients in the domains of understanding and appraising, and monitored patients to ensure they implemented the information:

Ah… she (my wife) does not allow. She is angry. Now you have diabetes still want to drink sweet tea. The condensed milk is dangerous. (P9)

## 4 DISCUSSION

This study aimed to explore the health literacy experiences of multi‐ethnic patients with T2DM. It highlighted that the experience of multi‐ethnic patients with regard to health literacy was not incorporated in previous conceptual models of health literacy. Health information is acquired through push and pull methods and trusted even without the need for appraisal. It also confirmed the importance of patients' motivation towards the development of health literacy skills.

There was a duality of experiences that occurred across the health literacy spectrum. Patients with adequate health literacy were motivated and actively looked for information to assist them in self‐management and to improve their diabetes understanding. It is difficult to postulate if these patients were motivated as a result of the information they obtained or that they obtained the information because they were motivated. Paashe‐Orlow and Wolf^26^ proposed that health literacy influences patient motivations which then leads to better health outcomes. Patients' experiences, captured in this study, suggest that motivation also played a role in enhancing their health literacy level by encouraging them to actively seek information. Health literacy and motivation may be separate but important concepts that influence patients' behaviour, much like health literacy and empowerment.^27^

In contrast, patients with limited health literacy received information pushed by people around them. These patients looked to HCPs as well as friends, family members and others who have T2DM for information. This confirmed the presence of a phenomenon called ‘distributed health literacy’ found by Edwards et al^28^ Distributed health literacy is a term coined to describe a situation where health literacy was found to be distributed through family and social networks, and patients with long‐term health conditions often drew on the health literacy skills of others to seek, understand and use health information. ^28^ Patients in this study also accessed information and benefited from the health literacy skills of their personal and community networks. Another important source of information for multi‐ethnic patients was their HCPs (the doctors or nurses). As reported by previous studies, doctors are seen as an authoritative source of diabetes knowledge and management^29^ and patients reported that ‘physicians were the best people to control glucose’.^30^ Patients, in this study, even viewed information giving as the responsibility of HCPs. Despite this trust, both health‐care professionals and patients reported language and communication issues as significant barriers in receiving and understanding information on diabetes management. Interpreter services in Malaysian health care are limited. Most translations in clinical settings were provided by HCPs and family members.

Information shared by individuals who are trusted by patients was accepted as it stands without being evaluated. Patients with adequate health literacy performed basic appraisal by comparing information between sources, while patients with limited health literacy often relied on their trust of those who conveyed the information. With the presence of an alternative pathway, patients often received conflicting information. Patients in this study noted that neither the HCPs nor their personal or community sources offered evidence to support their information. HCPs may want to discuss evidence and to impart the skills of information appraisal to their patients. Evidence evaluation skills could also be taught in schools.

### 4.1 Strengths

This study interviewed patients with adequate and limited health literacy and used a framework approach to analyse the data. Framework analysis is most suitable for analysis of interview data, where it is desirable to generate themes by making comparisons within and between cases.^25^ By purposively selecting patients with adequate and limited health literacy, this study was able to compare and contrast data across health literacy levels as well as within health literacy levels. Patients involved in the study were from all major ethnic groups in Malaysia and represented major ethnicities in Asia. They represented patients from varying backgrounds with different levels of social support and language proficiency. This study also captured the views of HCPs to triangulate the views and experiences shared by patients.

### 4.2 Limitations

One criticism is that the number of health‐care providers interviewed may not have achieved data saturation. However, we interviewed HCPs involved in the care of patients with T2DM in Malaysia such as family medicine specialists, diabetes educators and medical officers as a way of triangulating our findings. This study primarily set out to capture patients' experiences. Since health literacy is a function of not only the patient but also the health‐care systems that they access health care from, this study's findings mainly reflect patients with T2DM accessing care at primary care clinics.^31^

### 4.3 Implications for policy and practice

This study identified several points for intervention towards enhancing patients' health literacy levels. Adequate health literacy is essential for patient empowerment and involvement in their health care.^32^

Health‐care policymakers and providers need to be aware of current barriers faced by patients with T2DM and limited health literacy in order to deliver health care sensitive to patients' health literacy level. Interventions need to address the language barriers that are impeding patients' understanding as well as supporting HCPs who cited a lack of time and support. Currently, there are no such interventions implemented in Malaysia, but there are several interventions implemented globally that could serve as examples. In a 2010 systematic review of interventions targeting patients with T2DM and low health literacy, Van Scoyoc and DeWalt concluded that successful interventions combined personalized teaching and longitudinal follow‐up, and gave support to patients by helping them to overcome not only barriers to acquiring knowledge about diabetes but also personal and system barriers such as transportation and access to medications.^33^ By identifying the ways patients access information as well as their barriers to understanding, appraising and applying this information, results from this study could be used to tailor interventions aimed at improving health literacy and be directed to the patients, families, HCPs and the general population. Future research should aim to measure the impact of these interventions on patients' health literacy level and ultimately on diabetes outcomes.

## 5 CONCLUSION

This study found a duality in the health literacy experiences of multi‐ethnic patients with T2DM. These experiences were heavily influenced by culture, which explained the absence of this pathway in previous health literacy conceptual frameworks. Therefore, interventions to improve health literacy in these patients need to be targeted not just at patients but their families, friends, the general population and HCPs. Appropriate training must be provided to HCPs in view of the important role they play in improving patients' health literacy.

## CONFLICT OF INTEREST

The authors declare that they have no competing interests.

## ETHICAL APPROVAL

This study received ethics approval from the Medical Research and Ethics Committee of the Ministry of Health, Malaysia. (Approval Number: NMRR‐18‐2111‐42580 IIR).

## Supporting information

Appendix S1

Appendix S2

## Data Availability

The data that support the findings of this study are available from the corresponding author upon reasonable request.

## References

[1] Ratzan S , Parker RM . "Health literacy." National library of medicine current bibliographies in medicine. 2000 Bethesda: National Institutes of Health, US Department of Health and Human Services.

[2] Sørensen K , Van den Broucke S , Fullam J , et al. Health literacy and public health: A systematic review and integration of definitions and models. BMC Public Health. 2012;12(1):80. 22276600 10.1186/1471-2458-12-80 PMC3292515

[3] Schillinger D , Grumbach K , Piette J , Al E . Association of health literacy with diabetes outcomes. JAMA. 2002;288(4):475‐482. 12132978 10.1001/jama.288.4.475

[4] Chen GD , Huang CN , Yang YS , Lew‐Ting CY . Patient perception of understanding health education and instructions has moderating effect on glycemic control. BMC Public Health. 2014;14(1):683. 24996669 10.1186/1471-2458-14-683 PMC4094435

[5] Powell CK , Hill EG , Clancy DE . The relationship between health literacy and diabetes knowledge and readiness to take health actions. Diabetes Educ. 2007;33(1):144‐151. 17272800 10.1177/0145721706297452

[6] Caruso R , Magon A , Baroni I , et al. Health literacy in type 2 diabetes patients: a systematic review of systematic reviews. Acta Diabetol. 2018;55(1):1‐12. 29129000 10.1007/s00592-017-1071-1

[7] Thurston MM , Bourg CA , Phillips BB , Huston SA . Impact of health literacy level on aspects of medication nonadherence reported by underserved patients with type 2 diabetes. Diabetes Technol Ther. 2015;17(3):187‐193. 25531555 10.1089/dia.2014.0220

[8] Haun JN , Patel NR , French DD , Campbell RR , Bradham DD , Lapcevic WA . Association between health literacy and medical care costs in an integrated healthcare system: a regional population based study. BMC Health Serv Res. 2015;15(1):249. 26113118 10.1186/s12913-015-0887-z PMC4482196

[9] DeWalt DA , Boone RS , Pignone MP . Literacy and its relationship with self‐efficacy, trust, and participation in medical decision making. Am J Health Behav. 2007;31(SUPPL. 1):27‐35. 10.5555/ajhb.2007.31.supp.S27 17931133

[10] Ishikawa H , Yano E . The relationship of patient participation and diabetes outcomes for patients with high vs. low health literacy. Patient Educ Couns. 2011;84(3):393‐397. 21388773 10.1016/j.pec.2011.01.029

[11] Morris NS , Maclean CD , Littenberg B . Literacy and health outcomes: a cross‐sectional study in 1002 adults with diabetes. BMC Fam Pract. 2006;7(49):1‐8. 16907968 10.1186/1471-2296-7-49 PMC1559691

[12] Tang YH , Pang SMC , Chan MF , Yeung GSP , Yeung VTF . Health literacy, complication awareness, and diabetic control in patients with type 2 diabetes mellitus. J Adv Nurs. 2008;62(1):74‐83. 18352966 10.1111/j.1365-2648.2007.04526.x

[13] Kim SH , Lee A . Health‐literacy‐sensitive diabetes self‐management interventions: a systematic review and meta‐analysis. Worldviews Evidence‐Based Nurs. 2016;13(4):324‐333. 10.1111/wvn.12157 27104337

[14] Sheridan SL , Halpern DJ , Viera AJ , Berkman ND , Donahue KE , Crotty K . Interventions for individuals with low health literacy: a systematic review. J Health Commun. 2011;16(SUPPL. 3):30‐54. 21951242 10.1080/10810730.2011.604391

[15] Abdullah A , Liew SM , Salim H , Ng CJ , Chinna K . Prevalence of limited health literacy among patients with type 2 diabetes mellitus: a systematic review. PLoS One. 2019;14(5):1‐16. 10.1371/journal.pone.0216402 PMC6504081 31063470

[16] Kim SH . Health literacy and functional health status in Korean older adults. J Clin Nurs. 2009;18(16):2337‐2343. 19583664 10.1111/j.1365-2702.2008.02739.x

[17] Azreena E , Suriani I , Juni MH , Fuziah P . Factors associated with health literacy among Type 2 Diabetes Mellitus patients attending a government health clinic, 2016. Int J Public Heal Clin Sci. 2016;3(6):50‐64.

[18] Leung AYM , Bo A , Hsiao HY , Wang SS , Chi I . Health literacy issues in the care of Chinese American immigrants with diabetes: a qualitative study. BMJ Open. 2014;4(11):1‐12. 10.1136/bmjopen-2014-005294 PMC4244415 25406155

[19] Prabsangob K , Somrongthong R , Kumar R . Health literacy among Thai elderly population with type‐2 diabetes living in rural area of Thailand. Pakistan Journal of Public Health. 2018;8(1):27‐31.

[20] Bollars C , Sorensen K , de Vries N , Meertens R . Exploring health literacy in relation to noncommunicable diseases in Samoa: a qualitative study. BMC Public Health. 2019;19(1):1151. 31438907 10.1186/s12889-019-7474-x PMC6704563

[21] Andaya BW , Andaya LY . A History of Malaysia. London: MacMillan Press Ltd; 1982.

[22] Institute for Public Health (IPH) . National Health and Morbidity Survey 2015 (NHMS 2015). Vol. II: Non‐Communicable Diseases, Risk Factors & Other Health Problems; 2015.

[23] Bak Leong G . Loke Meng O , Yam Ngo L . 21st Report of the Malaysian Dialysis & Transplant Registry 2013; 2014.

[24] Mafauzy M , Hussein Z , Chan SP . The status of diabetes control in Malaysia: Results of DiabCare 2008. Med J Malaysia. 2011;66(3):175‐181. 22111435

[25] Gale NK , Heath G , Cameron E , Rashid S , Redwood S . Using the framework method for the analysis of qualitative data in multi‐disciplinary health research. BMC Med Res Methodol. 2013;13(1):1. 24047204 10.1186/1471-2288-13-117 PMC3848812

[26] Paasche‐Orlow MK , Wolf MS . The causal pathways linking health literacy to health outcomes. Am J Heal Behav. 2007;31(Suppl 1):19‐26. 10.5555/ajhb.2007.31.supp.S19 17931132

[27] Juul L , Rowlands G , Terkildsen H . Relationships between health literacy, motivation and diet and physical activity in people with type 2 diabetes participating in peer‐led support groups. Prim Care Diabetes. 2018;12:331‐337. 29559207 10.1016/j.pcd.2018.02.005

[28] Edwards M , Wood F , Davies M , Edwards A . “Distributed health literacy”: longitudinal qualitative analysis of the roles of health literacy mediators and social networks of people living with a long‐term health condition. Heal Expect. 2015;18(5):1180‐1193. 10.1111/hex.12093 PMC5060848 23773311

[29] Sohal T , Sohal P , King‐shier KM , Khan NA . Barriers and facilitators for type‐2 diabetes management in South Asians: a systematic review. PloS one. 2015;10(9):e0136202. 26383535 10.1371/journal.pone.0136202 PMC4575130

[30] Meetoo D , Meetoo L . Health promotion. Explanatory models of diabetes among Asian and Caucasian participants. Br J Nurs. 2005;14(3):154‐159. 15788935 10.12968/bjon.2005.14.3.17521

[31] Parker R , Jacobson K . Why is health literacy important? What do we know about health literacy? Efforts to improve quality, reduce disparities and reduce costs cannot succeed without improving health literacy. What Can Be Done? The Burden Does Not Lie Solely on the Individ. 2012.

[32] Protheroe J , Nutbeam D , Rowlands G . Health literacy: a necessity for increasing participation in health care. Br J Gen Pract. 2009;59(567):721‐723. 19843420 10.3399/bjgp09X472584 PMC2751916

[33] Van Scoyoc EE , Dewalt DA . Interventions to improve diabetes outcomes for people with low literacy and numeracy: a systematic literature review. Diabetes Spectr. 2010;23(4):228‐237.

