# Supplementary material for: Health literacy experiences of multi‐ethnic patients and their health‐care providers in the management of type 2 diabetes in Malaysia: A qualitative study
Source: Health Expect. 2020 Jul 19;23(5):1166–76. doi: 10.1111/hex.13095 (PMC7696131; doi:10.1111/hex.13095)
Supplement: Supplementary file 1 — Appendix S1 [file HEX-23-1166-s001.docx]

**Supplementary File S1: Topic guide for healthcare providers**

**Study title:** Health information behaviour in patients with type 2 diabetes mellitus (DM) in primary care: a qualitative study.

**Introduction:**

For the purposes of this study, you have been invited because you are directly or indirectly involved in the care of patients with type 2 DM in primary care. We are interested to capture your experience in giving health information to these patients.

Please also share with us your experience in helping patients to access, understand, appraise and apply health information they need for their diabetes management.

*Accessing Information*

- When faced with patients with type 2 DM, what were the questions they may ask you?
  - What information do you think they need to know?
- Can you share with me your experience in helping patients to find information?
  - What was your role?
  - How did you do it?
- Did you have any difficulties? What were they?
- Did you use any other resources to help you? Who or what?
- Were there times when you did not give patients particular information but they found it themselves and ask you about it? What was the information? How did they find it?

*Understanding Information*

- What do you think of the patients’ understanding of the information you provide? Or the information that they found themselves?
- How would you help patients to understand the health information received?
- Did you face any difficulties helping patients to understand the information received? What were they? What strategies did you take to deal with this?

*Appraising Information*

- What do you think of the patients’ assessment of the information you provide? Or the information that they found themselves?
- How would you help patients to assess the health information received?
- Did you face any difficulties helping patients to assess the information received? What were they? What strategies did you take to deal with this?

*Apply Information*

- What do you think of the patients’ ability to apply the information you provide? Or the information that they found themselves?
- How would you help patients to apply the health information received?
- Did you face any difficulties helping patients to apply the information received? What were they? What strategies did you take to deal with this?

• Is there anything else that you want to share with me?

~Thank You~
