# Supplementary material for: Health literacy experiences of multi‐ethnic patients and their health‐care providers in the management of type 2 diabetes in Malaysia: A qualitative study
Source: Health Expect. 2020 Jul 19;23(5):1166–76. doi: 10.1111/hex.13095 (PMC7696131; doi:10.1111/hex.13095)
Supplement: Supplementary file 2 — Appendix S2 [file HEX-23-1166-s002.docx]

**Supplementary File S2: Topic guide for patients with type 2 diabetes mellitus**

**Study title:** Health information behaviour in patients with type 2 diabetes mellitus (DM) in primary care: a qualitative study.

**Introduction:**

For the purposes of this study, you have been invited because you have diabetes and had used health information in order to manage your diabetes.

Please also share with us the your experience in accessing, understanding, appraising and applying health information needed to help you take care of your diabetes.

*Accessing Information*

- When faced with managing your diabetes, did you have any questions in mind?
  - What were they?
  - What did you try to find out? Why?
  - Were there any other reasons that makes you look for information on diabetes? What were the reasons?
- Can you share with me what you would do to get the answers to your questions/ to gather information?
  - e.g. ask your doctor/nurse, ask your family members or friends, internet, books, pamphlets etc. Why?
- Did you have any difficulties to get the answer? What were they?
- Do you have any help in your search for answers? Who or where?
- Were there times when you did not seek for the answer but the information was shared with you without you asking? Where/who shared the information? What information did they share?

*Understanding Information*

- Did you finally get an answer to all your questions? Was the information helpful?
- Did you face any difficulties to understand the information received? What were they?
- Did anyone or anything help you to further understand the information you found? How did it help?

*Appraising Information*

- Did you finally understand the information? Was the information helpful?
- How did you assess if the information you received is true and can be trusted?
- Did you face any difficulties to assess the information received? What were they?
- Did anyone or anything help you to further assess the information you found? How did it help?

*Apply Information*

- Did you finally use the information/answers that you received? Was the information helpful?
- How did the information/answers you had used in the management of your diabetes?
- Did you face any difficulties to apply the information received? What were they?
- Did anyone or anything help you to further understand the information you found? How did it help?

• Is there anything else that you want to share with me?

~Thank You~
